# Continuous Gastric pH Monitoring in Children Facilitates Better Understanding of Gastroesophageal Reflux Disease: A Prospective Observational Study

**DOI:** 10.3390/children12020236

**Published:** 2025-02-15

**Authors:** Shiva Sharma, Devendra I. Mehta, Nishant Patel, Arun Ajmera, Jeffrey Bornstein, Florence George

**Affiliations:** 1 Center for Digestive Health and Nutrition, Arnold Palmer Hospital for Children, Orlando, FL 32806, USA; devendra.mehta@orlandohealth.com (D.I.M.); nishant.patel@orlandohealth.com (N.P.); arun.ajmera@duke.edu (A.A.); jeffrey.bornstein@orlandohealth.com (J.B.); 2 Department of Mathematics & Statistics, Florida International University, Miami, FL 33199, USA; fgeorge@fiu.edu

**Keywords:** biliary reflux, duodenogastroesophageal reflux, histamine H2 receptor antagonists, multichannel intraluminal pH, non-acidic reflux, proton pump inhibitors

## Abstract

**Objectives:** Gastroesophageal reflux disease (GERD) is commonly encountered in adults and children. A subset of patients with GERD are refractory to acid suppressants, implicating other factors in the refluxate. Duodenogastric reflux (DGR) produces similar symptoms through reflux of non-acidic duodenal content and the cytotoxic effect of bile in the esophageal mucosa. Various methods have been utilized to detect DGR using a Bilitec device or Hepatobiliary scintigraphy, amongst the most common, each with their own limitations. We aimed to use combined multichannel intraluminal impedance and pH (MII-pH) monitoring with an additional gastric pH sensor to collect information about acidic and non-acidic gastroesophageal refluxes and to assess whether continuous gastric pH measurement in children provides indirect evidence of DGR for better understanding of the symptoms. **Methods:** From 2022 through 2023, clinically symptomatic pediatric patients scheduled for esophagogastroduodenoscopy (EGD) and MII-pH at Arnold Palmer Hospital for Children in the United States were included (n = 26). Exclusions included patients taking acid suppressants prior to the start of this study. The data were analyzed for subjects completing at least 18 h of the study protocol. **Results:** Subjects with a normal pH impedance (n = 5) showed a median non-meal gastric pH of 1.8. Subjects with an abnormal pH impedance (n = 21) showed a median non-meal gastric pH of 2.2. Of the 26 subjects enrolled, the duration of non-meal gastric pH 4.0–7.0 was positively correlated with non-acidic gastroesophageal refluxes. Although all acidic reflux events occurred at gastric pH < 4.0, there was no correlation between the duration of non-meal gastric pH < 4.0 and impedance changes or reflux index. **Conclusions:** The results showed daily variability in the non-meal gastric pH of pediatric patients and a statistically significant correlation between its duration at pH 4.0 to 7.0 and non-acidic refluxes suggestive of the implication of DGR. Further research is required to assess this association with gastroesophageal reflux and dyspeptic symptoms to investigate the diagnostic tools and therapeutic interventions, including the role of prokinetics and surface protective agents for DGR.

## 1. Introduction

### Background and Objectives

Pediatric gastroesophageal reflux disease (GERD) is common. Undetected, it may precipitate feeding intolerance, nausea, vomiting, abdominal pain, and lack of weight gain. There are ongoing efforts to optimize diagnostic tools and treatment options. Treatment includes two groups of acid suppressants: proton pump inhibitors (PPI) and histamine H2 receptor antagonists (H2RA). Some patients are refractory to acid suppressants, indicating that other factors in the refluxate, such as non-acidic reflux or duodenogastric reflux (DGR), may underlie the pathophysiology.

Esophageal pH monitoring is commonly used for diagnosis; however, combined multichannel intraluminal impedance and pH (MII-pH) monitoring provides specific reflux information: its extent in the esophagus; pH (acidic or non-acidic); composition (liquid, gas, or mixed); and the probability that refluxes are associated with symptoms [1,2,3,4,5,6,7,8].

A clear relationship exists between the degree and duration of acid suppression, relief from heartburn, and esophageal healing [9,10]. DGR, the retrograde passage of duodenal content into the stomach, is non-acidic (pH > 4) [11,12], yet studies in adults show an association between DGR and dyspeptic symptoms, and an increased prevalence of esophageal lesions [13,14]. Although DGR may result from certain surgical procedures (cholecystectomy, pyloroplasty, and gastric surgery), it also can result from pyloric insufficiency without secondary causes [15,16].

A small (n = 6) study of children symptomatic for GERD, but unresponsive to typical antacids, showed increased DGR responsive to therapy with prokinetics and surface protective agents [17]. Similarly, prokinetic was found to have beneficial influence on treatment results in children due to DGR [18]. Given the subsets of patients with reflux symptoms who are refractory to acid suppressants, along with the evolving evidence of association between DGR and dyspeptic symptoms, there is a need to reassess the role of non-acidic esophageal reflux in the evaluation of GERD.

## 2. Methods

### 2.1. Study Design and Population

From 2022 through 2023, subjects scheduled for endoscopy with pH impedance who exhibited symptoms suggestive of, or potentially related to GERD, were prospectively enrolled. As such, these subjects did not have any prior pH impedance study conducted. The average patient age was 7.53 years (n = 26, SD 4.2 years; range 1–18 years), with 61% male (n = 16). Patients’ history of acid suppressant or prokinetic use were documented at enrollment. The exclusion criteria included (i) subjects older than 18 years; (ii) those who had been treated with proton pump inhibitors (PPI) within 7 days prior to this study; (iii) those treated with histamine H2 receptor antagonist (H2A) or prokinetic agents within 5 days prior to this study; and (iv) study consent refusal from their caregiver/guardian.

### 2.2. Research Ethics Approval

This study was approved by the Institutional Review Board at Orlando Health Arnold Palmer Palmer Hospital for Children, IRB approval number 2210305, Orlando, Florida, United States of America.

### 2.3. Patient and Public Involvement

Neither the patients nor the public were involved in the design, conduct, reporting, or dissemination of the plans of this research.

### 2.4. Impedance–pH Measurement

Esophageal impedance, and esophageal and gastric pH monitoring were performed using ZandorpH single-use impedance and pH catheters, and the Ohmega™ ambulatory combined impedance–pH recorder (Laborie Medical Technologies Corporation, Portsmouth, NH, USA). The system includes an ambulatory impedance–pH recorder, a catheter containing two antimony pH electrodes (esophageal and gastric), and six impedance electrodes.

Subjects fasted overnight (approximately 8 h) prior to the procedure, and the catheters were calibrated using pH 4.0 and 7.0 buffer solutions before insertion. The catheter was inserted through the nostril and stabilized externally with surgical tape throughout the duration of the study (18–24 h). The gastric pH sensor was placed in the gastric body. The esophageal pH sensor was placed 3 cm above the lower esophageal sphincter (LES) for patients younger than 12 years and 5 cm above the LES for subjects 12 years and older. Accurate catheter placement was confirmed endoscopically, and subjects were either admitted for overnight observation or were discharged with instructions to resume normal activity, dietary, and sleep routines. Caregivers were provided a symptoms diary and instructed to record meals, symptoms, and postural changes throughout the study period.

### 2.5. Data Collection

**Figure 1:**
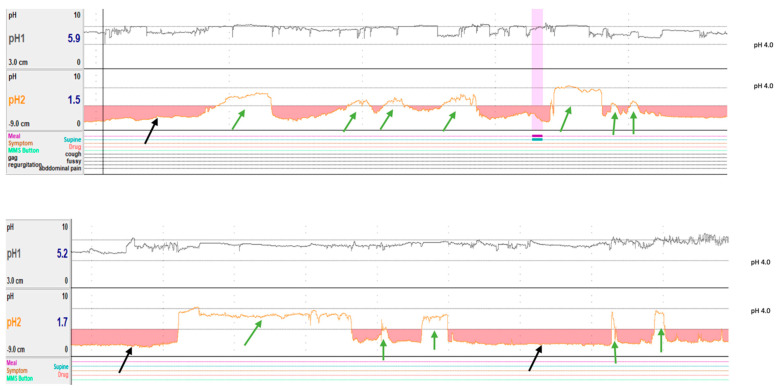
Examples of simultaneous non-meal esophageal (pH1) and gastric pH (pH2) monitoring traces from two randomly selected patients. Black arrows indicate intervals of acidic gastric pH. Green arrows indicate intervals of alkaline shift in gastric pH. Pink shade represents meal period.

Symptoms, meals, and postural position (supine/upright) were uploaded according to the patient diary. All reflux events were analyzed in supine and upright positions and during the day (8 a.m.–10 p.m.) and night (10 p.m.–8 a.m.). Meal durations were excluded from the data analysis. Two examiners manually identified and excluded potential artifacts (i.e., pseudo-reflux) and included missed reflux events. Non-meal gastric pH data were collected every 10 min for all patients. The data collected from the impedance and pH recordings included the total number of gastroesophageal refluxes (acidic or non-acidic); the reflux extent into the esophagus (proximal if extent of reflux between impedance sensors 1 and 3 or distal if extent of reflux between impedance sensor 4 and 6); the total gastric pH time (acidic or non-acidic) (Figure 1); the acid clearance time (time required for acid clearance from the esophagus); the reflux index (total percentage time esophagus was exposed to an acidic pH); the number of reflux periods > 2 min; and the longest reflux duration. The reflux parameters and methods of analyzing symptom associations are shown in Table 1.

### 2.6. Statistical Analyses

Data from the Ohmega ambulatory pH recorder were analyzed using SPSS^®^ v. 26 (IBM^®^ Corp, Armonk, NY, USA), with *p* < 0.05 (CI 95%) considered statistically significant. Simple linear regression and the Pearson correlation coefficient were used to examine the association between numeric variables. One-way analysis of variance (ANOVA) was used to compare the mean values among three or more data sets, and the *t* test was applied to compare the average of the two groups. For cases in which normal distribution of data was not met, we performed an appropriate non-parametric test.

## 3. Results

**Figure 2:**
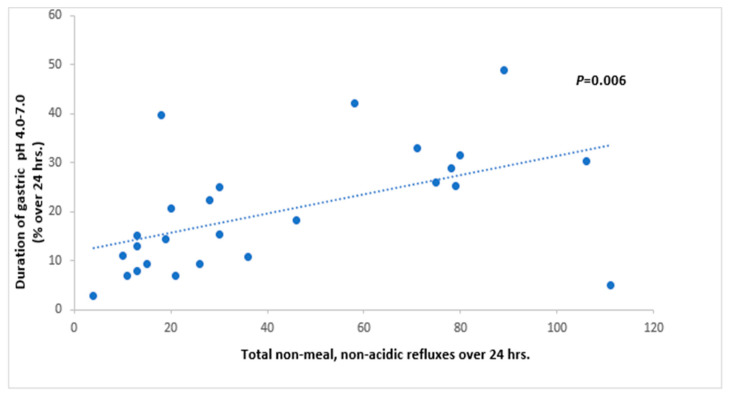
Association between non-meal, non-acidic esophageal refluxes and duration of non-acidic gastric pH. Datapoints represent total non-meal esophageal refluxes.

Table 2 shows non-meal gastric pH data for enrolled patients (n = 26). Table 3 shows the summary of non-meal esophageal refluxes, non-meal non-acidic gastric pH duration, and symptoms. The duration of gastric pH in the range between 4.0 and 7.0 was positively correlated with non-acidic esophageal refluxes (pH 4.0–7.0) (*p* = 0.006) (Figure 2) and with non-meal refluxes (*p* = 0.016). Although every esophageal acid reflux event, by definition, occurred when the gastric pH was <4.0, there was no correlation between the duration of gastric pH (<4.0) with impedance changes (*p* = 0.11) or with the reflux index (*p* = 0.16). Tissue biopsies showed unremarkable histology in 73% (n = 19) of patients. Reflux extent analyses showed that 14 (53%) subjects had proximal extent of reflux in the esophagus.

We did not find a significant association between the duration of gastric pH (4.0–7.0) with the following variables: (a) total number of long refluxes (>5 min) (*p* = 0.91); (b) age (*p* = 0.90); (c) extent of refluxes in the esophagus (*p* = 0.14); and (d) reflux index (*p* = 0.68). The median (IQR) number of documented symptoms per study period was 3 (1.0–8.5). We did not find a significant association between the duration of gastric pH > 4.0–7.0 and documented clinical symptoms.

## 4. Discussion

Duodenogastric reflux is defined as the retrograde passage of duodenal content into the stomach. Transient DGR is physiological in both adults and children [11,12]. Over time, this condition may progress to GERD that is non-responsive to acid suppressants [14,19]. Studies in adults with DGR confirm the cytotoxic effects of bile on the gastric epithelium, and increased histamine content and mast cell degranulation with subsequent mucosal injury [20]. The presence of duodenal refluxate in the esophagus has been implicated in impaired esophageal mucosal integrity and in the development of Barrett’s esophagus [19,21,22].

Various evaluation methods for DGR have been described in the literature, including (i) measuring intragastric bile acids with an ambulatory fiberoptic probe (commonly known as a Bilitec device) [23] and (ii) hepatobiliary scintigraphy [24]. Although intragastric monitoring has reliable sensitivity for DGR diagnosis in adults [25], this test is limited by the availability of the Bilitec device and the need for frequent intermittent sampling (hourly hand aspirates) of gastric contents. Hepatobiliary scintigraphy is of limited use due to short-term patient exposure to isotopes. The measurement of gastric contents and the analysis of phospholipids and trypsin have been studied as a modality to detect physiological DGR in children [12], yet pathological DGR criteria have not been established. Indirect DGR assessmentsenable us to assess symptoms associated with gastric pH changes over 24 h associated with sleep/wake cycles, meals, and supine or upright position. This is the first such study in a pediatric population, to our knowledge.

In the absence of true normal controls for this study, we used established impedance criteria to identify patients with a normal pH impedance study (n = 5) and those with an abnormal pH impedance study (n = 21). We found a positive correlation between the duration of gastric pH 4.0–7.0 and the number of non-acidic (pH 4.0–7.0) reflux episodes (*p* = 0.006) and the number of non-meal reflux episodes (*p* = 0.016). This correlation was not observed with the duration of gastric pH < 4.0. Since this correlation was observed during non-meal periods and with non-acidic gastric pH, these findings may suggest either a DGR or delayed gastric emptying, though the brief spike patterns of non-acidic gastric pH (Figure 1) seen are not consistent with delayed emptying. Given the unknown prevalence of gastroparesis in the pediatric population and relatively rare prevalence in young adults [26], DGR is a potential consideration for this observation and may warrant further research. Although there are various secondary causes of DGR (especially abdominal surgery), DGR can also develop primarily due to pyloric insufficiency [17]. We hypothesize there may be a shared mechanism underlying pyloric insufficiency and relaxation of the LES for the observation seen contributing to both DGR and GER at the same time. The majority of our patients did not show histological changes in the stomach, and this has been reported in only a few cases with DGR [18,24].

We did not find an association between the clinical symptoms and duration of non-acidic gastric pH, although the overall number of reported symptoms was too few to rule out an association.

## 5. Limitations

Our findings did not demonstrate a correlation between the duration of gastric pH < 4.0 with changes in impedance or with the reflux index, suggesting that DGR may play a significant role independent of the presence of a meal. However, the observations could also be influenced by the consumption of high-pH foods or undocumented mealtimes, which should be addressed in future studies. The subjects in this study did not have any underlying secondary causes suggestive of DGR, and specific studies for pyloric insufficiency were not performed. In addition, standard Ohmega software criteria recorded a positive GER association only with symptoms occurring within 2 min of reflux into the esophagus. This criterion does not apply to symptoms potentially related to DGR. A much larger population sample is required to correlate symptoms and to develop association criteria. As such, it is preliminary to accept or refuse the findings of this clinical correlation.

Further limitations include an obvious absence of true normal participants from this pediatric population, resulting in a small sample size of likely normal studies based on conventional impedance analysis. In addition, we did not document direct proof of duodenal refluxate in the stomach (e.g., by the Bilitec device, Hepatobiliary scintigraphy, or measurement of gastric contents for phospholipids and trypsin), given the invasive nature of this diagnostic procedure and the availability of these techniques.

## 6. Interpretation

Our study suggests the consideration for DGR in subjects with increased non-acidic GER. Consensus is lacking regarding the diagnostic modality for DGR and definition of pathological DGR. Given the episodic nature of the DGR, similar to GER, a diagnostic tool should be designed to monitor the DGR over a period of time similar to the esophageal impedance pH catheter. Few studies have reported the possible efficacy of prokinetics in mitigating DGR in children [17,18]. Therefore, studies in larger pediatric populations will be useful to develop specific diagnostic criteria, feasible investigational tools, and therapeutic options for pathological DGR in this population.

## Figures and Tables

**Table 1:** Definitions of reflux parameters and methods of analyzing symptom association in multichannel intraluminal impedance–pH monitoring.

Parameters	Definition
Liquid Reflux	Retrograde 50% impedance decline Begins distally (at the lower esophageal sphincter) Extends to at least 2 distal impedance sensors
Reflux	Acidic (pH < 4.0) Non-acidic (pH > 4.0)
Reflux Index	The total percentage of time of esophageal exposure to pH < 4
GER-associated Symptoms	Association determined by Ohmega software v2.04 Occurrence within 2 min of reflux onset
Symptom Index (SI)	% of reflux-associated symptoms divided by the total number of symptoms >50% = positive
Symptom Sensitivity Index (SSI)	% of symptom-associated reflux divided by the total number of refluxes >10% = positive
Symptom Association Probability (SAP)	Calculation of the statistical relation between reflux and symptoms Fisher’s extract test >95% = positive

**Table 2:** Non-meal gastric pH data for enrolled patients.

Group (pH Impedance Study)	Median IQR Gastric pH	8 a.m.–10 p.m.	10 p.m.–8 a.m.
Normal *	1.8 (1.4–2.6)	2.1 (1.6–3.4)	1.6 (1.3–2.0)
Abnormal	2.2 (1.2–3.8)	2.6 (1.8–4.3)	2.0 (1.6–2.7)

* Meets all three criteria: (1) reflux index < 10% for <1 yr of age and <5% for >1 yr of age. (2) Total impedance < 100 for <1 yr of age and <70 for >1 yr of age. (3) No symptom associations.

**Table 3:** Summary of esophageal refluxes, duration of non-acidic gastric pH, and symptoms.

Subject	Total Non-Meal Esophageal Refluxes	Total Non-Meal Acidic Esophageal Refluxes	Total Non-Meal Non-Acidic Esophageal Refluxes	Duration of Non-Acidic Gastric pH (% over 24 h)	Total Number of Reported Symptoms *	SAP > 95% (Yes/No)
1	29	7	22	6.9	0	No
2	45	16	29	22.4	0	Yes
3	139	26	113	4.9	1	Yes
4	103	24	79	25.3	1	Yes
5	147	41	106	30.3	1	No
6	15	4	11	6.8	1	Yes
7	95	14	81	28.9	1	No
8	88	69	19	14.3	1	No
9	46	31	15	9.4	2	No
10	59	6	58	18.1	2	Yes
11	103	26	77	26	2	Yes
12	8	4	4	2.8	3	No
13	21	8	13	15	3	Yes
14	21	7	14	13	3	No
15	15	2	13	7.9	4	No
16	49	18	31	9.4	5	No
17	25	13	12	10.9	5	No
18	97	38	59	42.2	7	No
19	37	16	21	20.7	7	No
20	123	52	71	33	9	Yes
21	19	1	18	39.8	9	No
22	96	60	36	10.7	10	Yes
23	106	16	90	48.8	14	Yes
24	141	61	80	31.5	16	Yes
25	46	3	43	25	17	No
26	73	43	30	15.3	40	No

* Cough, vomiting, regurgitation heartburn, chest pain, stomach pain, belching/burping, nausea.

## Data Availability

The data are available upon reasonable request to the corresponding author. The data are not publicly available due to privacy restrictions.
